# De novo design of selective compounds: a fragment-based pipeline applied to the β2 adrenergic receptor

**DOI:** 10.1186/1758-2946-6-S1-P25

**Published:** 2014-03-11

**Authors:** Florent Chevillard, Peter Kolb

**Affiliations:** 1 Kolblab, Institute of Pharmaceutical Chemistry, Philipps-University Marburg, Marbacher Weg 6-10, 35037 Marburg, Germany

## 

GPCRs play a key role in transmembrane signaling and are involved in many physiological processes, such as regulation of behavior, heart rate and the immune system. Therefore they are very important targets for pharmaceutical agents. Our project focuses on the β_2_ Adrenergic Receptor [1,2] (β_2_AR). The β_2_AR is mainly involved in vasodilation and bronchodilation in the human body. The recently solved structures of the β_2_AR open up new possibilities in the design of novel specific ligands using structure-based approaches. Here, we describe a pipeline to grow an unspecific fragment-sized scaffold for the β_2_AR. The protocol uses focused docking of fragments in two different zones identified within the binding site. The top ranked fragments are then computationally added to the core scaffold, filtered, minimized, evaluated by flexible ligand docking and inspected for later synthesis. Our initial results show that promising ligands can be identified by adding discriminating fragments to a core scaffold and that the generated compounds provide a reasonable synthetic accessibility.
